# EEG for Diagnosis of Autism Spectrum Disorder

**DOI:** 10.15844/pedneurbriefs-32-13

**Published:** 2018-11-09

**Authors:** Meghan O’Neill, Talia Shear

**Affiliations:** 1 Division of Developmental and Behavioral Pediatrics, Ann & Robert H. Lurie Children’s Hospital of Chicago, Chicago, IL; 2 Departments of Pediatrics and Neurology, Northwestern University Feinberg School of Medicine, Chicago, IL; 3 Division of Neurology, Ann & Robert H. Lurie Children’s Hospital of Chicago, Chicago, IL

**Keywords:** Autism, Autism Spectrum Disorder, EEG, Diagnosis, Early Diagnosis

## Abstract

Investigators from Boston University and the University of San Francisco studied whether EEG could be reliably used as an early biomarker for diagnosis of autism spectrum disorder (ASD).

Investigators from Boston University and the University of San Francisco studied whether EEG could be reliably used as an early biomarker for diagnosis of autism spectrum disorder (ASD). They enrolled 99 infant siblings of older children with a diagnosis of ASD ("high-risk" group), and 89 infants with no siblings or first-degree relatives with ASD ("low-risk" group) beginning at 3 months of age and continuing until 36 months. Ultimately, 3 children from the low-risk group and 32 from the high-risk group were diagnosed with ASD. Children were evaluated at several time points with EEG and the Autism Diagnostic Observation Schedule (ADOS), the current gold-standard clinical diagnostic tool which also quantifies severity of symptoms through the Calibrated Severity Score (CSS). The authors analyzed various features of the EEG data to create an algorithm to predict ASD diagnosis. They found that the EEG algorithm was highly predictive in distinguishing those children who were ultimately diagnosed with ASD from those who were not, at as early as 3 months of age. The EEG algorithm was able to differentiate the ASD and low-risk/no-autism group with 100% specificity and positive predictive value (PPV) at all ages of testing. Sensitivity in this group ranged from 82-100%. High-risk/no-autism infants were also distinguished from the ASD group at high rates but were generally more difficult to classify as their EEG data placed them near the diagnostic borderline. When EEG data from high and low risk non-autism groups were combined and compared to the ASD group, sensitivity ranged from 82-100%, specificity 88-99%, and PPV 65-97%. An overall dip in predictive accuracy was seen in both groups at 12 months of age, with improvement at and beyond 18 months. EEG findings were also used to predict severity of autism symptoms, with moderately strong correlation to actual CSS scores (r 0.45-0.55). [1]

COMMENTARY. This study’s results offer a promising future diagnostic possibility for early, reliable diagnosis of ASD. The institution of early autism-specific therapies is critical in supporting optimal development and is associated with better outcomes in ASD [2]. However, formal diagnosis prior to 3 years of age is challenging, as it is behaviorally, rather than biologically, diagnosed. Abnormal neural connectivity is thought to be associated with ASD and it has been suggested that neural network structure, a marker of neural connectivity, can be measured with EEG [3]. Prior research has shown that EEG features between high and low risk children are identifiably different and also change over time [4,5]. The current study expands upon this knowledge by including children ultimately diagnosed with ASD in their analysis, and by demonstrating that ASD diagnosis may be reliably predicted at as early as 3 months of age. The study also demonstrated the ability to quantify severity of autism symptoms, which may allow for monitoring of changing risk profiles during a child’s development and potentially even response to therapy. If larger studies confirm these results, EEG could serve as an early, non-invasive, relatively low-cost approach to early diagnosis of ASD and provide a basis for potentially initiating therapies at a much younger age.

## Disclosures

The author(s) have declared that no competing interests exist.
